# Mucus production stimulated by IFN-AhR signaling triggers hypoxia of COVID-19

**DOI:** 10.1038/s41422-020-00435-z

**Published:** 2020-11-06

**Authors:** Yuying Liu, Jiadi Lv, Jiangning Liu, Man Li, Jing Xie, Qi Lv, Wei Deng, Nannan Zhou, Yabo Zhou, Jiangping Song, Peng Wang, Chuan Qin, Wei-Min Tong, Bo Huang

**Affiliations:** 1 Department of Immunology & National Key Laboratory of Medical Molecular Biology, Institute of Basic Medical Sciences, Chinese Academy of Medical Sciences (CAMS) & Peking Union Medical College, Beijing, 100005 China; 2 Clinical Immunology Center, CAMS, Beijing, 100005 China; 3 NHC Key Laboratory of Human Disease Comparative Medicine, Beijing Key Laboratory for Animal Models of Emerging and Remerging Infectious Diseases, Institute of Laboratory Animal Science, CAMS and Comparative Medicine Center, Peking Union Medical College, Beijing, 100021 China; 4 Department of Pathology, Beijing Ditan Hospital, Capital Medical University, No.8 Jing Shun East Street, Chaoyang District, Beijing, 100015 China; 5 State Key Laboratory of Cardiovascular Disease, Fuwai Hospital, National Center for Cardiovascular Diseases, CAMS and Peking Union Medical College, 167A Beilishi Road, Xi Cheng District, Beijing, 100037 China; 6 Department of Pathology, Institute of Basic Medical Sciences, CAMS and Peking Union Medical College, Beijing, 100005 China; 7 Department of Biochemistry & Molecular Biology, Tongji Medical College, Huazhong University of Science & Technology, Wuhan, Hubei 430030 China

**Keywords:** Immunology, Innate immunity

## Abstract

Silent hypoxia has emerged as a unique feature of coronavirus disease 2019 (COVID-19). In this study, we show that mucins are accumulated in the bronchoalveolar lavage fluid (BALF) of COVID-19 patients and are upregulated in the lungs of severe respiratory syndrome coronavirus 2 (SARS-CoV-2)-infected mice and macaques. We find that induction of either interferon (IFN)-β or IFN-γ upon SARS-CoV-2 infection results in activation of aryl hydrocarbon receptor (AhR) signaling through an IDO-Kyn-dependent pathway, leading to transcriptional upregulation of the expression of mucins, both the secreted and membrane-bound, in alveolar epithelial cells. Consequently, accumulated alveolar mucus affects the blood-gas barrier, thus inducing hypoxia and diminishing lung capacity, which can be reversed by blocking AhR activity. These findings potentially explain the silent hypoxia formation in COVID-19 patients, and suggest a possible intervention strategy by targeting the AhR pathway.

## Introduction

The emergent outbreak of COVID-19 caused by SARS-CoV-2 has caused a global pandemic.^1,2^ Overall, acute respiratory disease syndromes (ARDS) are the major clinical symptoms; and COVID-19, upon entering a severe stage, becomes very difficult to manage and results in patient deaths.^3–5^ Currently, the decisive moment prompting death due to SARS-CoV-2 infection remains an enigma. It is generally believed that uncontrolled inflammation is involved in patient demise,^6,7^ and targeting the key proinflammatory cytokine IL-6 or JAK/STAT inflammatory pathway is already under investigation in critically ill patients with COVID-19.^7,8^ Overwhelming proinflammatory cytokines can damage alveolar epithelial and endothelial cells, leading to capillary permeability and pulmonary fibrinolysis, thus impeding the exchange of oxygen (O_2_) and carbon dioxide (CO_2_) and causing hypoxia, a key factor that initiates COVID-19-induced mortality.^9,10^ However, hypoxia seems to appear in the early stage of COVID-19, while excessive inflammation appears at a relatively late stage.^3,11^ Clinical practice has observed that many COVID-19 patients initially have oxygen deprivation without breathing problems,^12–14^ suggesting that other factor(s) prior to proinflammatory cytokines may contribute to COVID-19-induced hypoxia. Mucus coats the surfaces of cells lining the respiratory tract, which is vital to ensure trapping of inhaled particles and pathogens. Physiologically, mucus is ordinarily expelled out by the movement of cilia, which also prevents bronchiolar mucus from dropping into the lumens of alveoli where pneumocytes do not express cilium. However, mucus adherence and accumulation in the alveoli may increase the thickness of blood-gas barrier, thus hindering the exchange of O_2_ and CO_2_. Autopsy reports have revealed copious amounts of a gray-white viscous fluid in the lungs of COVID-19 patients.^15,16^ Consistently, protein exudates were also observed in a COVID-19 patient diagnosed following lung cancer surgery.^17^ In addition, a new study from single cell sequencing reported mucin expression in lung epithelial cells of COVID-19 patients.^18^ These observations prompted us to hypothesize that SARS-CoV-2 infection stimulates mucus production, thus promoting hypoxia by hindering O_2_ diffusion at the alveolar sites.

## Results

### Mucus is produced in lung epithelial cells of SARS-CoV-2-infected patients and macaques

**Fig. 1:**
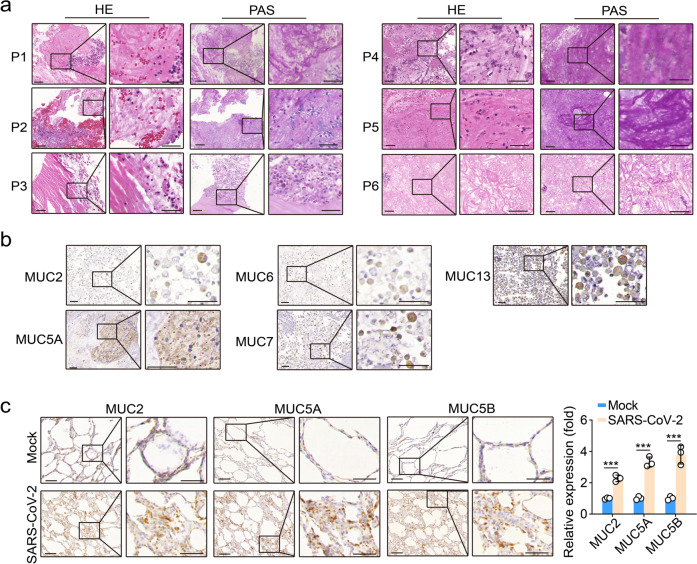
Mucins are expressed in COVID-19 patients and nonhuman macaques. **a** H&E and periodic acid-Schiff (PAS) staining of bronchoalveolar lavages (BALs) from COVID-19 patients. Scale bars, 50 μm. **b** Immunohistochemical staining for mucins 2, 5A, 6, 7 and 13 in the BAL from COVID-19 patients. Scale bars, 50 μm. **c** Immunohistochemical staining for mucins 2, 5A, and 5B from the lung sections of macaques infected with SARS-CoV-2 for 7 days (*n* = 3 macaques/group). The data represent means ± SD. Data (**a**, **b**) are representative images of 8 COVID-19 patients. Scale bars, 50 μm. ***P < 0.001, by two-tailed Student’s *t* test (**c**).

Bronchitis in the inferior lobe of the lung and a dry cough are common in COVID-19 patients,^3^ suggesting that SARS-CoV-2 invades the lung epithelial cells in the lower conducting airways and alveolar regions. Accordingly, if SARS-CoV-2 stimulates mucus production, mucus present in the alveoli should be found in bronchoalveolar lavages (BALs). Therefore, paraffin-embedded BALs for pathological diagnosis were collected from eight COVID-19 patients. Mucus is acidic and the hematoxylin-eosin staining showed that extracellular components were stained red (Fig. 1a). The viscosity of mucus lies in the linked oligosaccharides, which can be stained by periodic acid-Schiff (PAS). As expected, PAS staining showed abundant carbohydrates in BALs (Fig. 1a), suggesting that mucus-like substance might be present in the BAL of COVID-19 patients. Mucins are the major component of the mucus. Thus, we conducted additional immunochemical staining and a panel of mucins including the secreted gel-forming mucins 2, 5A, 6 and 7 and the membrane-tethered mucin 13 were each identified in the BAL (Fig. 1b). In line with this result, the expression of mucins 2, 5A and 5B were remarkably upregulated in SARS-CoV-2 infected macaques (Fig. 1c). Together, these results suggest that mucus is likely to be produced in the lung epithelial cells of COVID-19 patients.

### Mucins are upregulated by IFNs in alveolar epithelial cells

**Fig. 2:**
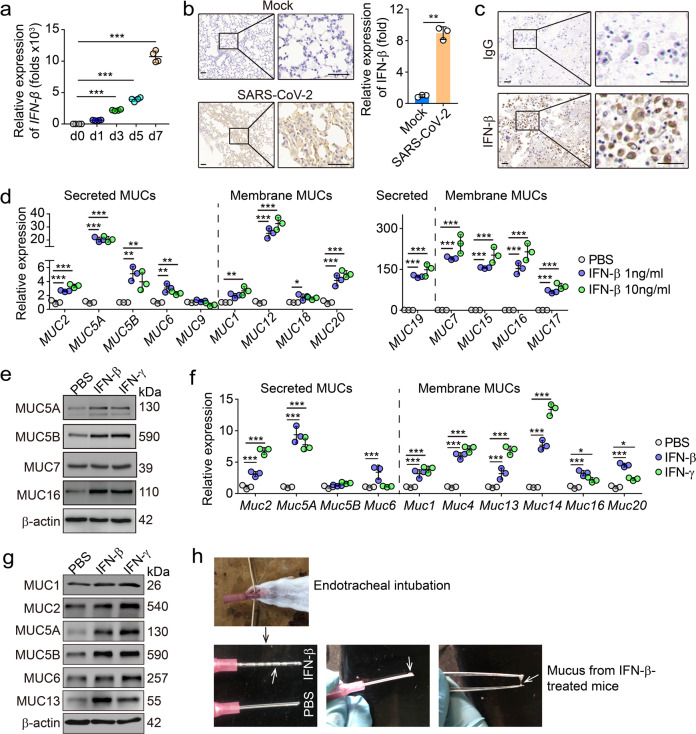
Both IFN-β and IFN-γ upregulate the expression of mucins. **a** hACE2-transgenic mice were infected with SARS-CoV-2 for the indicated time period. The expression of *IFN-β* was measured by real-time PCR. **b** Immunohistochemical staining of IFN-β from the lung sections of SARS-CoV-2-infected hACE2-transgenic mice for 5 days. Scale bars, 50 μm. **c** Representative image of immunohistochemical staining of isotype control-IgG or IFN-β in BAL from three COVID-19 patients. Scale bars, 50 μm. **d** BEAS-2B cells were treated with different doses of IFN-β (1 or 10 ng/mL) for 24 h. The expression of secreted mucins (*MUCs 2*, *5A*, *5B*, *6*, *9* and *19*) and membrane-mucins (*MUCs 1*, *7*, *12*, *15*, *16*, *17*, *18* and *20*) was determined by real-time PCR. **e** Western blot analysis of the expression of mucins 5A, 5B, 7 and 16 from BEAS-2B cells treated with PBS, IFN-β (1 ng/mL) or IFN-γ (10 ng/mL) for 48 h. **f** The same as (**d**), except that primary alveolar epithelial cells (PAECs) were treated with IFN-β (1 ng/mL) or IFN-γ (10 ng/mL). **g** Institute of Cancer Research (ICR) mice were treated with IFN-β (1 μg/mouse) or IFN-γ (10 μg/mouse) through the trachea once every day for 4 days. The expression of mucins 1, 2, 5A, 5B, 6 and 13 from the lung tissues was determined by western blot. **h** The same as (**g**), except the mucus was shown from the tracheal tubes. White arrow indicated the mucus. The data represent means ± SD. Representative images are from three mice (**b**, **e**, **g** and **h**), four mice (**a**) or three independent experiments (**d** and **f**). *P < 0.05, **P < 0.01, ***P < 0.001, by one-way ANOVA (**a**, **d** and **f**) or two-tailed Student’s *t*-test (**b**).

**Fig. 3:**
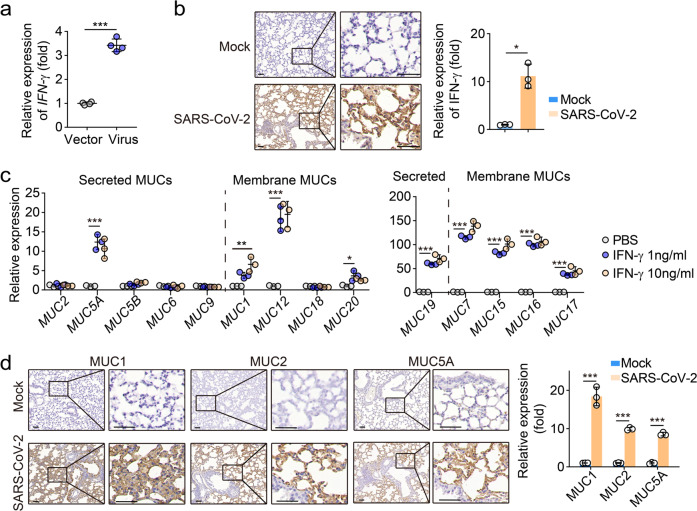
The expression of mucins was highly increased in SARS-CoV-2-infected mice. **a**, **b** hACE2-transgenic mice were infected with SARS-CoV-2. The expression of IFN-γ was detected at day 7 after SARS-CoV-2 infection by real-time PCR (**a**) or immunohistochemical staining with anti-IFN-γ antibody (**b**). Scale bars, 50 μm. **c** BEAS-2B cells were treated with different doses of IFN-γ (1 or 10 ng/mL) for 24 h. The expression of secreted mucins (*MUCs 2*, *5A*, *5B*, *6*, *9* and *19*) and membrane-mucins (*MUCs 1*, *7*, *12*, *15*, *16*, *17*, *18* and *20*) was determined by real-time PCR. **d** The same as (**b**), except that the expression of mucins 1, 2 and 5A was determined. Scale bars, 50 μm. The data represent means ± SD. Representative images are from three mice (**b** and **d**), four mice (**a**) or three biological independent samples (**c**). **P < 0.01, ***P < 0.001, by two-tailed Student’s *t*-test (**a**, **b** and **d**) or one-way ANOVA (**c**).

Next, we investigated the molecular pathway by which SARS-CoV-2 infection triggered mucin expression. A possibility is that SARS-CoV-2 may enter and directly stimulate lung epithelial cells to express mucins. Human lung epithelial cell line BEAS-2B was angiotensin-converting enzyme 2 (ACE2) positive^19^ and could be infected by SARS-CoV-2 (Supplementary information, Fig. S1a–d). However, the incubation of SARS-CoV-2 particles with BEAS-2B cells didn’t result in the upregulation but even decreased the expression of mucins, as evidenced by real time PCR (Supplementary information, Fig. S1e), suggesting an alternative way to induce mucin expression during SARS-CoV-2 infection. It is well known that type I IFN is the first alarm in response to viral invasion, whose signaling activates a set of genes, including protein kinase R and Mx proteins, in order to exert direct antiviral effects.^20^ Indeed, IFN-β expression was strikingly upregulated in a time-dependent manner in SARS-CoV-2-infected hACE2-transgenic mice (Fig. 2a, b). In addition, a moderate IFN-β expression was also observed in BALs of COVID-19 patients (Fig. 2c). Then, we treated BEAS-2B cells with IFN-β. We found that IFN-β treatment upregulated the expression of mucins 1, 2, 5A, 5B, 6, 7, 12 and 15–20 at both mRNA and protein levels (Fig. 2d, e). Moreover, we also found that primary mouse alveolar epithelial cells expressed high levels of mucins upon IFN-β treatment (Fig. 2f). In line with these results, the inhalation of IFN-β elevated the production of mucins in the lungs of mice (Fig. 2g, h), suggesting that IFN-β is capable of inducing lung epithelial cells to produce mucins. In addition to type I IFN, IFN-γ, which is pivotal for antiviral T cell immunity, is also increased in the lungs of SARS-CoV-2-infected transgenic mice (Fig. 3a, b). We further demonstrated that those secreted form or membrane-bound mucins could be induced by IFN-γ (Figs. 2e–g, 3c). This result was further supported by elevated levels of mucins 1, 2, 5A, 5B and 18 in the lung tissues from SARS-CoV-2-infected hACE2-transgenic mice (Fig. 3d; Supplementary information, Fig. S2a). Using mucins 1, 5A and 5B as representative, we also found that these mucins were co-localized with surfactant protein C (SPC), a marker for type II alveolar epithelial cells (Supplementary information, Fig. S2b). Given that IL-6 is highly elevated during SARS-CoV-2 infection,^21,22^ we additionally used IL-6 to treat primary lung epithelial cells. However, we found that IL-6 did not upregulate mucin expression in the cells (Supplementary information, Fig. S2c). Together, these results suggest that lung epithelial cells can be induced to upregulate mucin expression by SARS-CoV-2 infection-induced IFNs.

### IFNs induce mucin expression through an IDO/AhR-dependent pathway

**Fig. 4:**
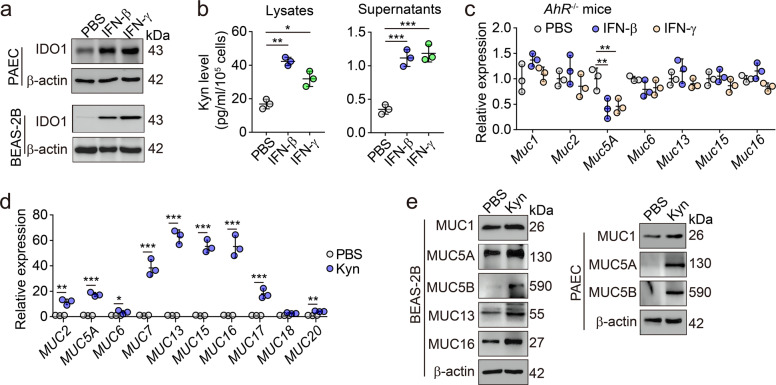
IFN-β and IFN-γ activated the Kyn-AhR pathway for mucin expression. **a** PAECs or BEAS-2B cells were treated with IFN-β (1 ng/mL) or IFN-γ (10 ng/mL) for 48 h. The expression of IDO1 was determined by western blotting. **b** BEAS-2B cells were treated with IFN-β (1 ng/mL) or IFN-γ (10 ng/mL) for 48 h. The levels of Kyn from cell lysates or the supernatants were determined by ELISA. **c** PAECs were isolated from *AhR*^–/–^ mice were treated with IFN-β (1 ng/mL) or IFN-γ (10 ng/mL) for 24 h. The expression of *Mucs 1*, *2*, *5A*, *6*, *13*, *15* and *16* was determined by real-time PCR. **d** BEAS-2B cells were treated with Kyn (400 μM) for 24 h. The expression of mucins (*MUCs 2*, *5A*, *6*, *7*, *13*, *15*, *16*, *17*, *18* and *20*) was determined by real-time PCR. **e** Western blot analysis of the expression of mucins 1, 5A, 5B, 13 and 16 from BEAS-2B cells or PAECs treated with Kyn (400 μM) for 48 h. The data represent means ± SD. In **b**–**d**, *n* = 3 biological independent samples. Representative images are from three independent experiments (**a** and **e**). *P < 0.05, **P < 0.01, ***P < 0.001, by two-tailed Student’s *t*-test (**d**) or one-way ANOVA (**b** and **c**).

**Fig. 5:**
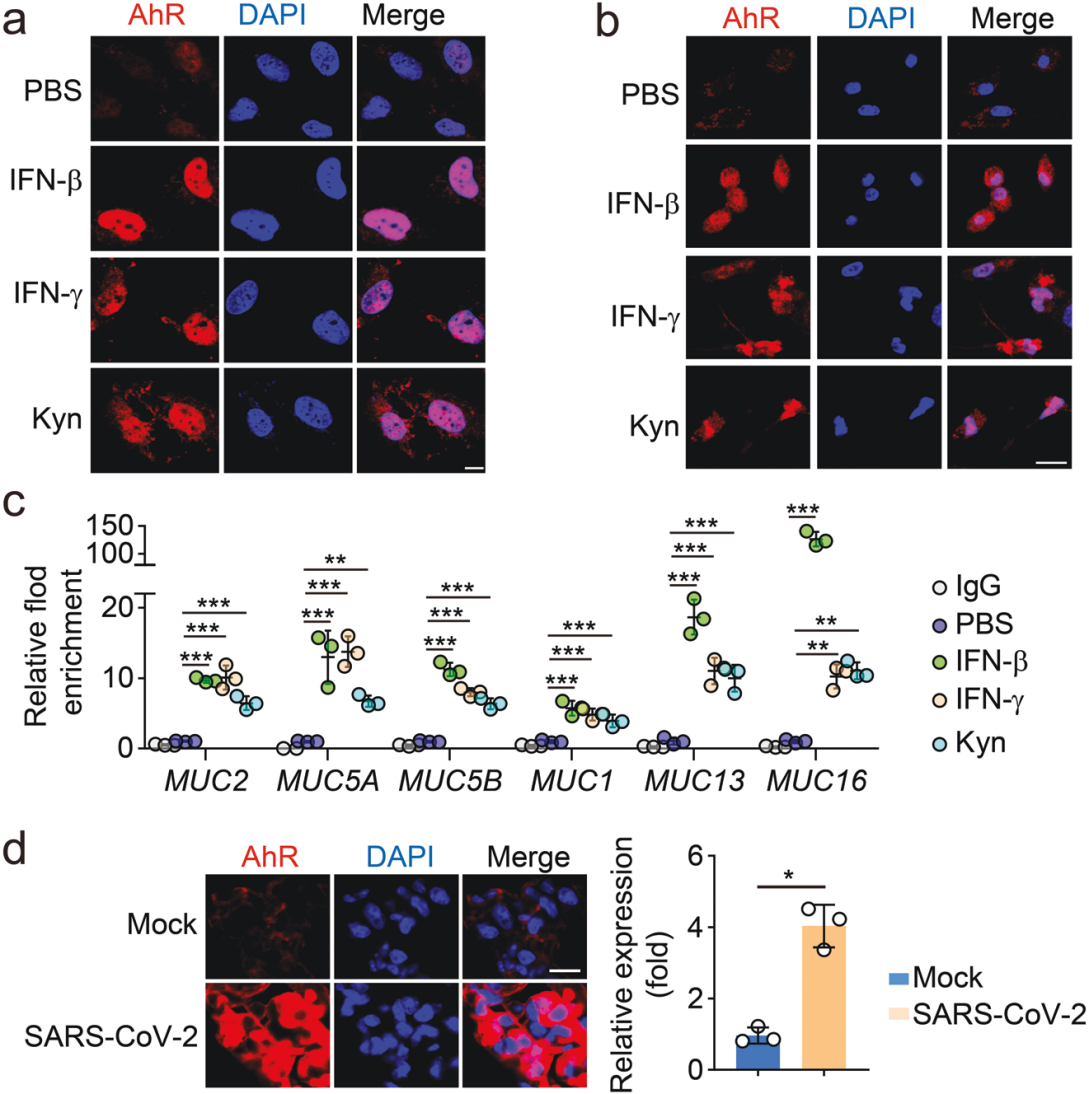
AhR-mucins pathway was activated. **a**, **b** BEAS-2B cells (**a**) or PAECs (**b**) were treated with IFN-β (1 ng/mL), IFN-γ (10 ng/mL) or Kyn (400 μM) for 48 h. Cells were immunostained with an anti-AhR antibody and observed under a confocal microscope. Scale bar, 10 μm. **c** BEAS-2B cells were treated with IFN-β (1 ng/mL), IFN-γ (10 ng/mL) or Kyn (400 μM) for 48 h. ChIP-qPCR analysis was performed with anti-AhR antibody and *MUCs* 2, 5A, 5B, 1, 13 and 16 promoter-specific primers. Data are present as relative fold enrichment to the PBS group. **d** hACE2-transgenic mice were infected with SARS-CoV-2 and the expression and localization of AhR (red) from the lung tissues was observed under confocal microscope. Scale bar, 10 μm. The data represent means ± SD. In **c**, *n* = 3 biological independent samples. Representative images are from three independent experiments (**a** and **b**) or three mice (**d**). *P < 0.05, **P < 0.01, ***P < 0.001, by two-tailed Student’s *t*-test (**d**) or one-way ANOVA (**c**).

Then, we explored the molecular mechanism by which IFNs regulated the expression of mucins. Upon binding to receptors, IFN-β and IFN-γ activate classical STAT2 and STAT1 signaling pathways, respectively. Additionally, IFN-β and IFN-γ can also activate a cytoplasmic transcription factor aryl hydrocarbon receptor (AhR).^23–25^ In our recent studies, we found that IFN-β and IFN-γ both are able to activate AhR through the indoleamine-pyrrole 2,3-dioxygenase (IDO)-Kynurenine (Kyn) pathway.^23–25^ In detail, IFN-activated STAT1 can upregulate IDO expression, thus triggering the Kyn-AhR signaling pathway.^24,25^ As an enzyme, IDO catalyzes the conversion of tryptophan to Kyn, an endogenous ligand for AhR.^26^ We found that IDO levels were upregulated by IFNs in lung epithelial cells (Fig. 4a), simultaneous with Kyn production (Fig. 4b). As a critical cytoplasmic transcription factor, AhR translocates into the nucleus upon activation and regulates gene expression through binding to a specific DNA sequence called dioxin-responsive element (DRE).^27^ We found that the addition of AhR inhibitor CH223191 blocked the effect of IFNs on mucin expression in both BEAS-2B and primary lung epithelial cells (Supplementary information, Fig. S3a, b). Furthermore, IFNs almost completely lost their effect on mucin expression in the AhR^–/–^ primary cells (Fig. 4c). Like IFNs, Kyn, the ligand for AhR, was also able to upregulate the expression of mucins in BEAS-2B cells as well as in isolated murine lung alveolar epithelial cells (Fig. 4d, e). Following IFNs or Kyn stimulation, the translocation of AhR into the nucleus of the cells was observed (Fig. 5a, b). Subsequently, the binding of AhR to the promoter of multiple mucin genes (MUC1, 2, 5 A, 5B, 13 and 16) was verified by chromatin immunoprecipitation-quantitative polymerase chain reaction (ChIP-qPCR) (Fig. 5c). Using the hACE2-transgenic mice as a SARS-CoV-2 infection model, AhR nuclear translocation was also confirmed (Fig. 5d). Notably, this nuclear translocation seemed to be correlated with the levels of IFN-β. Following SARS-CoV-2 challenge, the mRNA quantity of IFN-β was gradually elevated in the lungs of transgenic mice (Fig. 2a), parallel with the increased entry of AhR into the nucleus and the augmented mRNA expression of mucins (Supplementary information, Fig. S4a, b). Together, these results suggest that IFNs induce mucin expression in lung epithelial cells through an IDO/AhR-dependent pathway.

### AhR blockade ameliorates lung pathology in infected mice

**Fig. 6:**
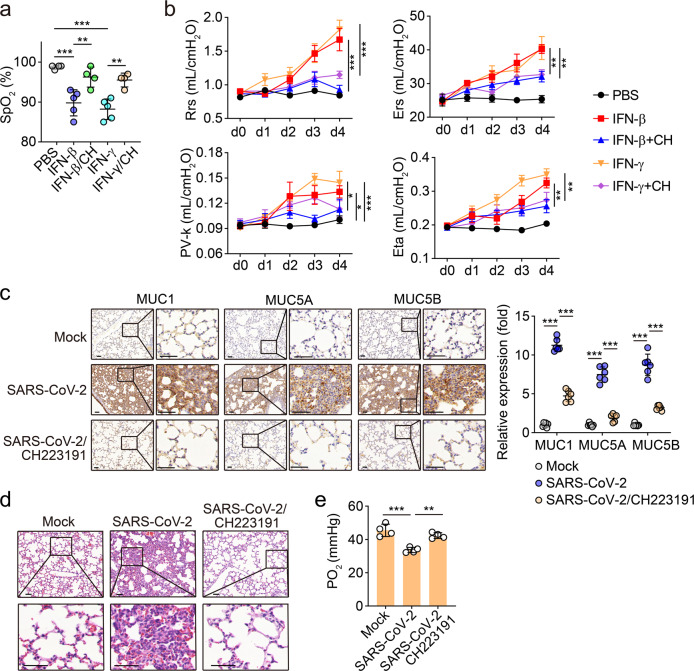
AhR inhibition reversed the aggravated lung function induced by IFN-β or IFN-γ. **a**, **b** ICR mice were treated with IFN-β (1 μg/mouse), IFN-γ (10 μg/mouse), IFN-β + CH223191 (10 mg/kg) or IFN-γ + CH223191 through the trachea once every day for 4 days. Blood oxygen saturation was measured at day 5 after treatment (**a**). Rrs (resistance of the respiratory system), Ers (elastance of the respiratory system), PV-k (curvature of the upper portion of the deflation limb of the Pressure/volume loop) and Eta (tissue hysteresivity) were detected at indicated time points (**b**). *n* = 5 mice. **c**–**e** hACE2-transgenic mice were infected with SARS-CoV-2, and then treated with or without CH223191 (10 mg/kg) by tail vein once every day for 5 days. The lung tissues were fixed to perform the immunohistochemical staining of mucins 1, 5A and 5B (**c**, *n* = 6 mice) and H&E staining (**d**, *n* = 6 mice). The PO_2_ was also analyzed (**e**, *n* = 4 mice). Scale bars, 50 μm. The data represent means ± SD. *P < 0.05, **P < 0.01, ***P < 0.001, by one-way ANOVA (**a**–**c** and **e**).

Finally, we investigated whether AhR activation-induced mucus resulted in hypoxia and diminished lung capacity. Type I IFN has been reported to recruit and activate innate immune cells that produce high levels of inflammatory mediators,^28^ thus promoting inflammatory damage of tissues. Here, we used IFN-β or IFN-γ to treat the mice through a bronchial atomization method, and found that IFN-β and IFN-γ were able to decrease partial pressure of O2 (SpO_2_) (Fig. 6a), leading to hypoxia, and likewise increase Rrs (resistance of the respiratory system), Ers (elastance of the respiratory system), PV-k (curvature of the upper portion of the deflation limb of the Pressure/volume loop) and Eta (tissue hysteresivity) (Fig. 6b), indicating impaired lung function of the mice. Then, we blocked the AhR pathway by intravenous injection of AhR inhibitor into the mice. Under this condition, the IFN-β- or IFN-γ-induced impairment of respiratory function was abrogated in the mice (Fig. 6a, b). In line with this result, AhR blockade also inhibited the expression of mucins in the lungs of the IFN-treated mice (Supplementary information, Fig. S5a, b). Moreover, we infected hACE2-transgenic mice with SARS-CoV-2, followed by CH223191 treatment once per day. We found that 5-day treatment resulted in a marked decrease of mucins 1, 4, 5A, 5B and 16 (Fig. 6c; Supplementary information, Fig. S5c), and reduced the pathological damage in the lungs (Fig. 6d). In line with these results, we found that the CH223191 treatment could reverse the decreased PO_2_ of peripheral blood induced by SARS-CoV-2 infection (Fig. 6e). Together, these results suggest that AhR blockade ameliorates lung pathology in SARS-CoV-2-infected mice.

## Discussion

**Fig. 7:**
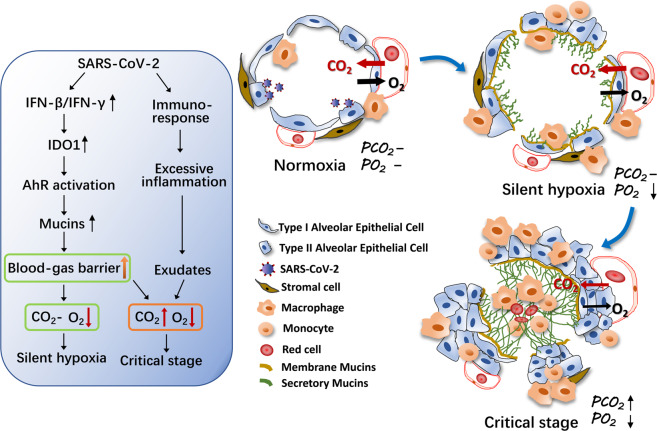
A schematic for AhR-upregulated mucins in the hypoxia of COVID-19 patients. A normal gas exchange between the alveoli and pulmonary capillary blood is achieved through a passive diffusion of O_2_ and CO_2_. During SARS-CoV-2 infection, increased IFN-β and IFN-γ activate the AhR pathway, leading to the expression of mucins in alveolar epithelial cells. Mucins then stick to the blood–gas barrier and increase the thickness. Thickened barrier hinders O_2_ crossing but not CO_2_ at the beginning, leading to a clinical symptom of silent hypoxia. Companying the disease progression, more mucin production in combination with inflammation-induced exudate further increases barrier thickness, impeding the exchange of both O_2_ and CO_2_, leading to a critical illness. “–”, normal level.

The complexity of COVID-19 lies in the fact that patients may not present initial symptom, but once symptoms appear, the illness progresses rapidly. The underlying mechanism might be elucidated by this study. In the lungs, O_2_ is inhaled from air but CO_2_ is generated from tissue cells through the tricarboxylic acid (TCA) cycle. As a waste product, CO_2_ is released into tissue interstitial fluid and further diffuses into capillary vessels. Subsequently, it is expelled through the lungs during exhalation. The body may be more tolerant to hypoxia but is very sensitive to higher partial pressure of CO_2_ (PCO_2_), because CO_2_ accumulation can lead to acidemia and cellular energy dysbolism. Physiologically, O_2_ and CO_2_ exchange between the alveoli and pulmonary capillary blood is achieved through a passive diffusion process (normoxia, Fig. 7), which can be influenced by thickness of the blood–gas barrier and solubility of the gas. In this study, mucus in either membrane-bound or secreted form, produced by alveolar epithelial cells in the alveoli of COVID-19 patients or SARS-CoV-2-infected hACE2-transgenic mice or rhesus macaques, can adhere to the gas–blood barrier and increase the thickness, thus hindering gas diffusion. Although O_2_ and CO_2_ face the same increased thickness, their solubility is different. CO_2_ has 20-fold higher solubility than O_2_.^29^ Thus, at an early stage of SARS-CoV-2 infection, an increased mucus-based barrier may only influence O_2_ diffusion but without affecting CO_2_ diffusion. As long as CO_2_ can be expelled, the blood pH homeostasis can be maintained and the mitochondrial TCA cycle can be normally processed and maintain a threshold-exceeding energy metabolism. Thus, COVID-19 patients, although experiencing hypoxia, can maintain a relative physiological homeostasis without presenting overt symptoms, clinically called silent hypoxia (Fig. 7).^12–14,30^ Once the disease progresses, mucus production increases, either secreted form or membrane-bound, along with inflammation-induced extrudate in alveoli, and interstitial inflammatory cell responses, which further increases the thickness of blood–gas barrier and results in the impediment of CO_2_ diffusion. Thus, at this point, COVID-19 patients can rapidly develop acidosis and cellular TCA cycle hinderance, leading to a critically ill stage and even death (Fig. 7). Indeed, clinical observation has found that critically ill patients with normal PCO_2_ can be rescued, while it is very difficult to rescue patients with altered PCO_2_.^31^ Therefore, targeting IFN-triggered AhR pathway might be a potential strategy to effectively treat COVID-19 patients.

An important finding in this study is that mucins can be expressed by alveolar epithelial cells in response to SARS-CoV-2 infection. Cells at the site of respiratory tract always meet and are invaded by pathogens. To defend this invasion, cells produce mucins to prevent pathogens from physical contact. On the other hand, IFN signals are the basic and first-line signals, which initiate a rapid antiviral response in cells. It is reasonable that at the beginning of viral invasion, immune cells first meet the virus and are triggered to release IFNs including IFN-β and IFN-γ. Such IFNs can deliver the viral invasion message to nearby tissue cells and make them to produce mucins, thus preventing viral attack in advance. In our previous studies,^23–25^ we have demonstrated that IFNs activate AhR, consistent with other studies.^32,33^ In addition, it has been shown that AhR activation in airway epithelial cells induced mucin 5AC.^34^ Thus, the activation of AhR by IFNs may stimulate alveolar epithelial cells to produce mucus. In addition, mucins are physiologically produced by goblet cells rather than airway ciliated epithelial cells or other cells.^35^ In bronchi and bronchioles, goblet cells secrete mucus and the ciliated cells move the mucus, thus expelling entrapped contaminants from the airway.^36^ The alveolus is a tiny, thin-walled, capillary-rich sac structure where the exchange of O_2_ and CO_2_ takes place. Type I and type II pneumocytes, which are lined in the alveoli, neither express cilia nor produce mucus, thus facilitating a dry and clean state of the alveoli. Biologically, this is a strategy used by cells to prevent themselves from viral attack, and cells may not care the negative impact of the mucus on the alveolar function. A puzzle in this study is that in BEAS-2B cells, we did not find the expression of mucins following SARS-CoV-2 infection. In theory, viral infection may cause cells to produce IFNs through a viral PAMP-activated signaling pathway. As a result, the released IFNs can activate the IDO-Kyn-AhR pathway, thus resulting in the upregulation of mucin expression. A possible explanation is that SARS-CoV-2 is very efficient at antagonizing IFN signaling in BEAS-2B cells.^37–39^

Given that the production of IFNs is a ubiquitous host response following viral infection, the blockade of the AhR pathway to reduce mucin expression by epithelial cells may also be suitable to treat various viral infection-caused lung disorders such as SARS, MERS and Ebola. In addition, influenza virus infection pervades during the spring and autumn seasons, which, although also activating the IFN-AhR pathway, does not cause the COVID-19-like severe illness. This might be due to that influenza virus usually does not invade the alveoli.^40^ Thus, the locally produced IFNs only have effects on bronchial or bronchiolar epithelial cells and resultant mucins can be cleared by ciliary movement. However, AhR blockade might be also useful for severe flu. This aspect is worthy of further investigation.

## Materials and methods

### Animals and cell lines

Female Institute of Cancer Research (ICR) mice, 6–8 weeks, were purchased from the Center of Medical Experimental Animals of the Chinese Academy of Medical Science (Beijing, China). AhR^–/–^ C57BL/6 mice were presented by Dr. Jun Yan (Third Military Medical University). These animals were maintained in the Animal Facilities of the Chinese Academy of Medical Science under specific pathogen-free conditions. hACE2-transgenic mice (6–11 months) with specific pathogen-free and rhesus macaques (3–4 years old) were obtained from the Institute of Laboratory Animal Science, Peking Union Medical College. Animals studies involving SARS-CoV-2 were performed in an animal biosafety level 3 (BASL3) facility using HEPA-filtered isolators and the procedures were approved by the Institutional Animal Care and Use Committee of the Institute of Laboratory Animal Science, Peking Union Medical College (BLL20001). Murine studies without viral infection were approved by the Animal Care and Use Committee of the Chinese Academy of Medical Science. Normal human bronchial epithelium cell line BEAS-2B cells were purchased from the China Center for Type Culture Collection (Shanghai, China) and cultured in RPMI1640 medium (Gibco, USA) or DMEM medium (Gibco, USA) with 10% FBS.

### Human samples

The paraffin embedding BALs of COVID-19 patients were obtained from the Department of Pathology, Beijing Ditan Hospital. Ethical review was granted by the Institutional Ethics Committee of Beijing Ditan Hospital (2020–025–01). The BALs from four males and four females, age from 22 to 82 years old, were used for cytological and histological diagnosis for COVID-19. The clinical features of the patients are listed in Supplementary information, Table S1.

### Reagents

Kynurenine was from Sigma-Aldrich (ST, USA). IFN-β and IFN-γ were purchased from PeproTech (Rocky Hill, NJ). Fludarabine (STAT1 inhibitor) and CH223191 were purchased from Selleck (Beijing, China).

### Isolation of primary alveolar epithelial cells type II

Primary alveolar epithelial cells type II were isolated from ICR mice or AhR^–/–^ mice as previously reported.^41,42^ Briefly, mice were perfused with 10 mL cold PBS through the right ventricle. Lungs were filled with 2 mL dispase (BD Bioscience, CA, USA) and low gelling temperature agarose (Sigma Aldrich, MO, USA) before lung tissues were incubated with 2 mL dispase in 37 °C for 20 min. Then, lung tissues were rubbed and the slurry was filtered through 70- and 40-μm nylon meshes (JETBIOFIL, China). The cells suspension was incubated with biotinylated anti-CD45 (Biolegend, clone 30-F11, Cat. 103104), anti-CD16/32 (BD Pharmingen^TM^, clone 2.4G2, Cat. 553143), anti-CD31 (Biolegend, clone MEC13.3, Cat. 102504), anti-TER119 (Biolegend, clone TER119, Cat. 116204) and anti-CD104 (Biolegend, clone 346–11A, Cat. 123603) antibodies at 4 °C for 30 min and then Dynabeads^TM^ MyOne^TM^ streptavidin T1 magnetic beads (Thermo Fisher Scientific, Cat. 65601) were added to the cell suspension to exclude leukocytes, monocytes/macrophages, NK cells, neutrophils, endothelial cells and erythroid cells. Negative selection of fibroblasts was performed by adherence on non-coated plastic plates. Cell purity was assessed routinely by flow cytometry.

### Western blotting

Cells were collected, lysed in M2 lysis buffer and sonicated. The protein concentrations were determined by a BCA kit (Applygen Technologies Inc., China). Then, the protein was run on a SDS-PAGE gel and transferred to nitrocellulose. Nitrocellulose membranes were blocked in 5% bovine serum albumin (BSA) and probed with antibodies overnight: anti-actin (Cell Signaling, Cat. 3700; 1:1,000); anti-human IDO1 (Cell Signaling, Cat. 86630; 1:1,000); anti-mouse IDO1 (Merck, Cat. 05–840; 1:1,000); anti-mucin 1 (Abcam, Cat. ab45167; 1:1,000); anti-mucin 2 (Abcam, Cat. ab272692; 1:1,000); anti-mucin 5A (Abcam, Cat. ab24071; 1:100); anti-mucin 5B (Abcam, Cat. ab77995; 1:1,000); anti-mucin 6 (Abcam, Cat. ab192318; 1:1,000); anti-mucin 7 (Abcam, Cat. ab105466; 1:1,000); anti-mucin 13 (Abcam, Cat. ab124654; 1:1,000) or anti-mucin 16 (Abcam, Cat. ab1107; 1:1,000). Secondary antibodies conjugated to horseradish peroxidase were followed by enhanced chemiluminescence (Thermo fisher, MA). Results were confirmed by at least three independent experiments.

### Immunofluorescence

Cells were fixed in 4% paraformaldehyde and permeabilized with 0.2% Triton X-100. For lung sections, the tissues were fixed in 10% formalin. Fixed cells or lung tissues were blocked in 5% BSA and incubated with anti-AhR (GeneTex, Cat. GTX129013; 1:1,000), mucin 1(Abcam, Cat. ab45167, 1:1000), mucin 5A (Abcam, Cat. ab24071, 1:100), mucin 5B (Abcam, Cat. ab77995, 1:1000), SPC (Abcam, Cat. ab211326, 1:1000) or SARS-CoV-2-S (Abcam, Cat. ab273433, 1:200) at 4 °C overnight. Then, cells or lung sections were washed and incubated with secondary antibodies for 1 h at room temperature. Finally, the slides were counterstained with DAPI and mounted for confocal analysis. The intensity of immunofluorescence was analyzed by Image J 9.0 software.

### Histological and immunohistochemical (H&E) staining

The lung tisses from mice or the BAL from COVID-19 patients were fixed in in 10% formalin, embedded in paraffin and sectioned for H&E staining. Immunohistochemical staining was performed using the DAB Horseradish Peroxidase Color Development Kit (ZSGB-BIO, China) according to the manufacturer’s instructions. Briefly, the sections of paraffin embedded tissues were incubated with anti-mucin 1 (Abcam, Cat. ab45167; 1:1,000), anti-mucin 2 (Abcam, Cat. ab272692; 1:1,000), anti-mucin 5A (Abcam, Cat. ab24071; 1:100), anti-mucin 5B (Abcam, Cat. ab77995; 1:1,000), anti- mucin 6 (Abcam, Cat. ab212648; 1:500), anti-mucin 7 (Abcam, Cat. ab105466; 1:1,000), anti-mucin 13 (Abcam, Cat. ab124654; 1:200), anti-mucin 18 (Abcam, Cat. ab75769; 1:400), anti-IFN-β (Abcam, Cat. ab180616; 1:200) or anti-IFN-γ (Abcam, Cat. ab218426; 1:200) at 4 °C overnight. Afterwards, slides were sequentially incubated with HRP-conjugated secondary antibodies for one hour at room temprature. For fluorescent staining, the slides were incubated with PANO Reagent PPD520 or PDD570 using the PANO 4-plex IHC Kit (Panovue, China) according to the manufacturer’s instructions, followed by conterstaining with DAPI (Thermo, USA) and finally mounting for confocal analysis. The stained lung sections were scanned and digitalized utilizing a TissueFaxs Plus System coupled onto a Zeiss Axio Imager Z2 microscope and Nikon A1 confocal microscope. The intensity of positive staining was analyzed by Image J 9.0 software.

### Real-time PCR

Total RNA was extracted from cells using Trizol (Invitrogen) and was transcribed to cDNA by using a high capacity cDNA reverse transcription kit (Applied Biosystems, CA). The primer sequences are shown as follows: h*β-actin*, 5′-CATGTACGTTGCTATCCAGGC-3′ (sense) and 5′-CTCCTTAATGTCACGCACGAT-3′ (antisense); *MUC1*, 5′-TGCCGCCGAAAGAACTACG-3′ (sense) and 5′-TGGGGTACTCGCTCATAGGAT-3′ (antisense); *MUC2*, 5′-GGAGATCACCAATGACTGCGA-3′ (sense) and 5′-GAATCGTTGTGGTCACCCTTG-3′ (antisense); *MUC5A*, 5′-CAGCACAACCCCTGTTTCAAA-3′ (sense) and 5′-GCGCACAGAGGATGACA GT-3′ (antisense); *MUC5B*, 5′-GCCCACATCTCCACCTATGAT-3′ (sense) and 5′-GCAGTTCTCGTTGTCCGTCA-3′ (antisense); *MUC6*, 5′-GTGTACGACTTCTCGGGGAC-3′ (sense) and 5′-TTGCTGGTATAGGGCAGGCT-3′ (antisense); *MUC7*, 5′-CACCAGAAGCCGTTCATTAGAA-3′ (sense) and 5′-GGGTTGACCACACTGCTATTT-3′ (antisense); *MUC9*, 5′-TCCCACACATCGTCCAAACAT-3′ (sense) and 5′-TCCCCATGATGAGCTTCTCTG-3′ (antisense); *MUC12*, 5′-CCAGTTCAAGCGACCCTTTTA-3′ (sense) and 5′-CGCTGTGGGATACTGTTGATT-3′ (antisense); *MUC13*, 5′-ATGCGTGCTGATGACAAGTTT-3′ (sense) and 5′-ACACCGAAGGGTCAAATCATAGT-3′ (antisense); *MUC15*, 5′-TATTCACTTCTATCGGGGAGCC-3′ (sense) and 5′-GGGAATGACTCGCCTTGAGAT-3′ (antisense); *MUC16*, 5′-CCAGTCCTACATCTTCGGTTGT-3′ (sense) and 5′-AGGGTAGTTCCTAGAGGGAGTT-3′ (antisense); *MUC17*, 5′-CCAACCCCAGCTTATAGTGAAG-3′ (sense) and 5′-GACCACAAGCGTAGTGCTGA-3′ (antisense); *MUC18*, 5′-AGCTCCGCGTCTACAAAGC-3′ (sense) and 5′-CTACACAGGTAGCGACCTCC-3′ (antisense); *MUC19*, 5′-TCAGAACAGGCAATACCCCAG-3′ (sense) and 5′-TGGAAGGACCTGAGATGGAAG-3′ (antisense); *MUC20*, 5′-GAGACCTCTTCTAGGGCCTCA-3′ (sense) and 5′-CACCATGAAGTTGGGAGATGTT-3′ (antisense); m*β-actin*, 5′-GGCTGTATTCCCCTCCATCG-3′ (sense) and 5′-CCAGTTGGTAACAATGCCATGT-3′ (antisense); *Muc1*, 5′-CGTCAGGCTCAGCTATCATTC-3′ (sense) and 5′-GGGTATTGACTTGGCACTGAA-3′ (antisense); *Muc2*, 5′-ATGCCCACCTCCTCAAAGAC-3′ (sense) and 5′-GTAGTTTCCGTTGGAACAGTGAA-3′ (antisense); *Muc3*, 5′-GCCGTGAATTGTATGAACGGA-3′ (sense) and 5′-CGCAGTTGACCACGTTGACTA-3′ (antisense); *Muc4*, 5′-CCTCCTCTTGCTACCTGATGC-3′ (sense) and 5′-GGAACTTGGAGTATCCCTTGTTG-3′ (antisense); *Muc5A*, 5′-CAGGACTCTCTGAAATCGTACCA-3′ (sense) and 5′-GAAGGCTCGTACCACAGGG-3′ (antisense); *Muc5B*, 5′-GTGGCCTTGCTCATGGTGT-3′ (sense) and 5′-CGCTCATGCTAGGGAAGACAG-3′ (antisense); *Muc6*, 5′-CACCTTTGACGGCCATGAGTA-3′ (sense) and 5′-GGTGTAGGGCAGGCTAACAA-3′ (antisense); *Muc13*, 5′-GATCTCTGCAACCCTAACCCC-3′ (sense) and 5′-TCCTTTCACACATGACGACAG-3′ (antisense); *Muc14*, 5′-AATACCAGGCATCGTGTCAGT-3′ (sense) and 5′-CCACTTCATGTTTTGGTGTTGTC-3′ (antisense); *Muc15*, 5′-ACCTGGCTAATGACTAGCTCA-3′ (sense) and 5′-AGACCTGTAAAGGATTTGCGTC-3′ (antisense); *Muc16*, 5′-CTCCACAGTGGTCACTCTTGA-3′ (sense) and 5′-AGTGGGAGGAGGCATTCAGT-3′ (antisense); *Muc20*, 5′-GCAAACTCTCAGCACTGAATCT-3′ (sense) and 5′-AGTGGTAGTATGTGTCTTCCTGG-3′ (antisense); *Cyp1a1*, 5′-GACCCTTACAAGTATTTGGTCGT-3′ (sense) and 5′-GGTATCCAGAGCCAGTAACCT-3′ (antisense); *Cyp1b1*, 5′-CACCAGCCTTAGTGCAGACAG-3′ (sense) and 5′-GAGGACCACGGTTTCCGTTG-3′ (antisense); SARS-CoV-2, 5′-GGGGAACTTCTCCTGCTAGAAT-3′ (sense) and 5′-CAGACATTTTGCTCTCAAGCTG-3′ (antisense). Real-time PCR was performed using ABI step-one plus (Applied Biosystems, CA, USA). Values are means ± SD from three independent experiments which were performed in duplicate. Statistical comparisons among groups were performed using a Student’s *t*-test. Values of all parameters were considered statistically significant difference at a value of *P* < 0.05.

### ChIP-qPCR

ChIP-qPCR was performed by using a MAGnity^TM^ Chromatin Immunoprecipitation System (Invitrogen, USA) according to the manufacturer’s protocol. In brief, BEAS-2B cells were crosslinked and chromatin was extracted and sheared. Samples were immunoprecipitated with anti-AhR antibody (Cell Signaling, USA). The primer sequences used for ChIP-qPCR are shown as follows: *MUC1*, 5′-TGCCACGCCCGATCTGCCTCT-3′ (sense) and 5′-GAACGCACAGCATGGTGG-3′ (antisense); *MUC2*, 5′-TCTGCCACCCGGTCGGCT-3′ (sense) and 5′-GGCACTGGTGGTGGTCGTCTG-3′ (antisense); *MUC5A*, 5′-TTCAACCCTAACCCCTCTGC-3′ (sense) and 5′-TGTGTGATGAACACTTGCCCAG-3′ (antisense); *MUC5B*, 5′-CTGCTTCCTCAACCCTGCC-3′ (sense) and 5′-GCCTTCCTCTATGTCTTGCCT-3′ (antisense); *MUC13*, 5′-CTTCAGCCTGGGCGACAGAGTGAG-3′ (sense) and 5′-ACCAGCTTTACTGCTTGGATT-3′ (antisense); *MUC16*, 5′-ACAAAAACAGCCAGAGATAGT-3′ (sense) and 5′-CGGAGGTTGCAGTGAGCCAAG-3′ (antisense). The results were from three independent experiments followed by normalization to input signals and shown as means ± SD.

### Lung function detection

Lung function measurement was performed according to the procedure previously published.^43^ In brief, mice were injected intraperitoneally (i.p.) with 0.4 g/kg tribromoethanol and placed on a flexiVent pulmonary system (SCIREQ Inc., Canada). Then, the mice were subjected to mechanical ventilation with a tidal volume of 10 mL/kg and a respiratory rate of 150 breaths/min. 3 cmH_2_O positive end expiratory pressure (PEEP) was used for lung function detection. The dynamic pulmonary compliance was measured by Snapshot-150 perturbation.

### Animal experiments and treatment protocol

ICR mice were treated with IFN-β (1 μg/mouse), IFN-γ (10 μg/mouse), IFN-β + CH223191 (10 mg/kg) or IFN-γ + CH223191 through the trachea once every day for 4 days. For SARS-CoV-2 infection, hACE2-transgenic mice were inoculated intranasally with PBS or SARS-CoV-2 stock virus at a dose of 1 × 10^5^ TCID_50_ for indicated time periods.

Rhesus macaques (3–4 years old) were challenged with 10^6^ TCID_50_/mL SARS-CoV-2 virus through the trachea (*n* = 3 macaques/group). At 7 days post inoculation, macaques were euthanized and lung tissues were collected for immunohistochemical staining.

### Partial pressure of O_2_ (PO_2_) analysis

Mice were anesthetized and the blood was drawn from canthus vein. Immediately, the level of PO_2_ was detected by using Roche Cobas b123 full-automatic blood gas analyzer (Roche).

### Quantification and statistical analysis

All experiments were performed at least three times. Results are expressed as means ± SD as indicated, and analyzed by two-tailed Student’s *t*-test or One-way ANOVA followed by Boferroni’s test. The *P* value < 0.05 was considered statistically significant. The analysis was conducted using the Graphpad 8.0 software.

## Supplementary information

Supplementary Figure S1

Supplementary Figure S2

Supplementary Figure S3

Supplementary Figure S4

Supplementary Figure S5

Supplementary Table

## Data Availability

All data needed to evaluate the conclusions in the paper are present in the paper or the Supplementary Materials. Materials described in the study are available either commercially or upon request from the corresponding author.
