# Supplementary material for: Mucus production stimulated by IFN-AhR signaling triggers hypoxia of COVID-19
Source: Cell Res. 2020 Nov 6;30(12):1078–87. doi: 10.1038/s41422-020-00435-z (PMC7646495; doi:10.1038/s41422-020-00435-z)
Supplement: Supplementary file 1 — Supplementary Figure S1 [file 41422_2020_435_MOESM1_ESM.pdf]

Fig. S1

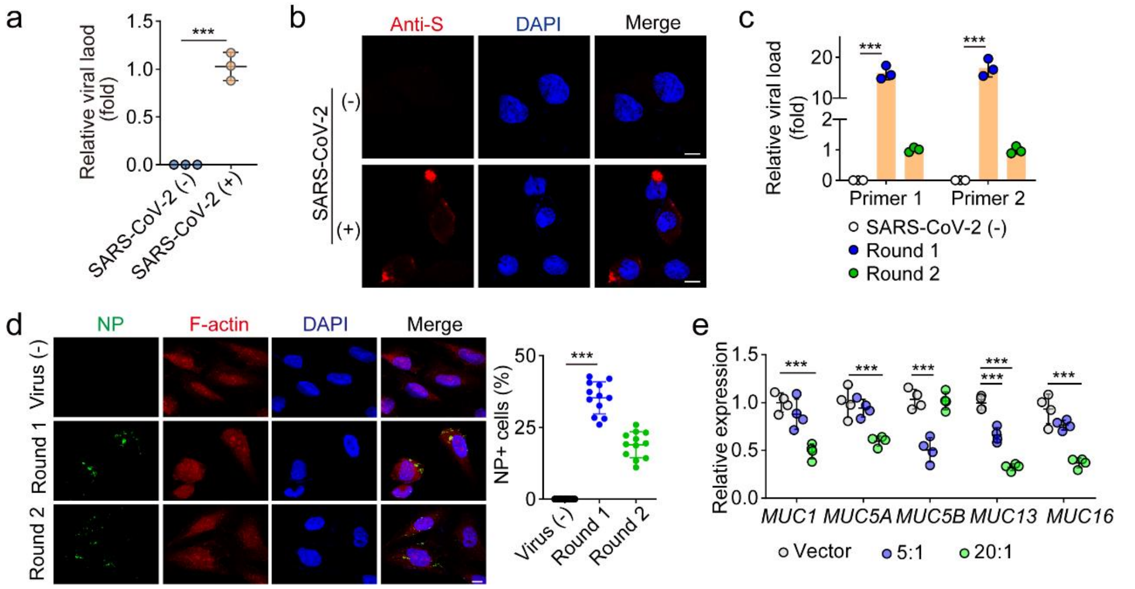

Fig. S1: SARS-CoV-2 cannot directly upregulate the expression of mucins. a BEAS-2B cells were infected with or without SARS-CoV-2 at the ratio of 5:1 ($TCID_{50}$: cells) for 24 hr. The expression of SARS-CoV-2 was determined by real-time PCR. **b** BEAS-2B cells were infected with or without SARS-CoV-2 at the ratio of 5:1 ($TCID_{50}$: cells) for 2 hr. Cells were fixed and immunostained with anti-SARS-CoV-2-S antibody. Scale bar, 10 μm. **c, d** BEAS-2B cells (1×10^5 cells) were infected with SARS-CoV-2 (5×10^5 $TCID_{50}$) for 8 hr, and then cultured in virus-free medium for another 40 hr. The supernatants were collected and concentrated. Uninfected-BEAS-2B cells were treated with the above concentrated supernatants for 48 hr. Some cells were fixed to stain with anti-NP (nucleocapsid protein for SARS-CoV-2, green color) or F-actin (red color) antibody (Round 1) and observed by confocal microscope (**d**). Other cells were collected for RNA extraction (Round 1) (**c**). The supernatants were collected, concentrated and added into another uninfected-BEAS-2B cells for 48 hr. These cells were either fixed for immunostaining or performed the real-time PCR analysis (Round

2). Scale bar, 10 μm. **e** BEAS-2B cells were infected with SARS-Cov-2 at the ratio of 5:1 or 20:1 for 24 hr. The expression of *MUCs 1*, *5A*, *5B*, *13* and *16* was determined by real-time PCR. The data represent mean ± SD. n = 3 biological independent samples. *** $P<0.001$, by two-tailed Student's t test (**a**) or by one-way ANOVA (**c**-**e**).
