# Supplementary material for: Mucus production stimulated by IFN-AhR signaling triggers hypoxia of COVID-19
Source: Cell Res. 2020 Nov 6;30(12):1078–87. doi: 10.1038/s41422-020-00435-z (PMC7646495; doi:10.1038/s41422-020-00435-z)
Supplement: Supplementary file 2 — Supplementary Figure S2 [file 41422_2020_435_MOESM2_ESM.pdf]

Fig. S2

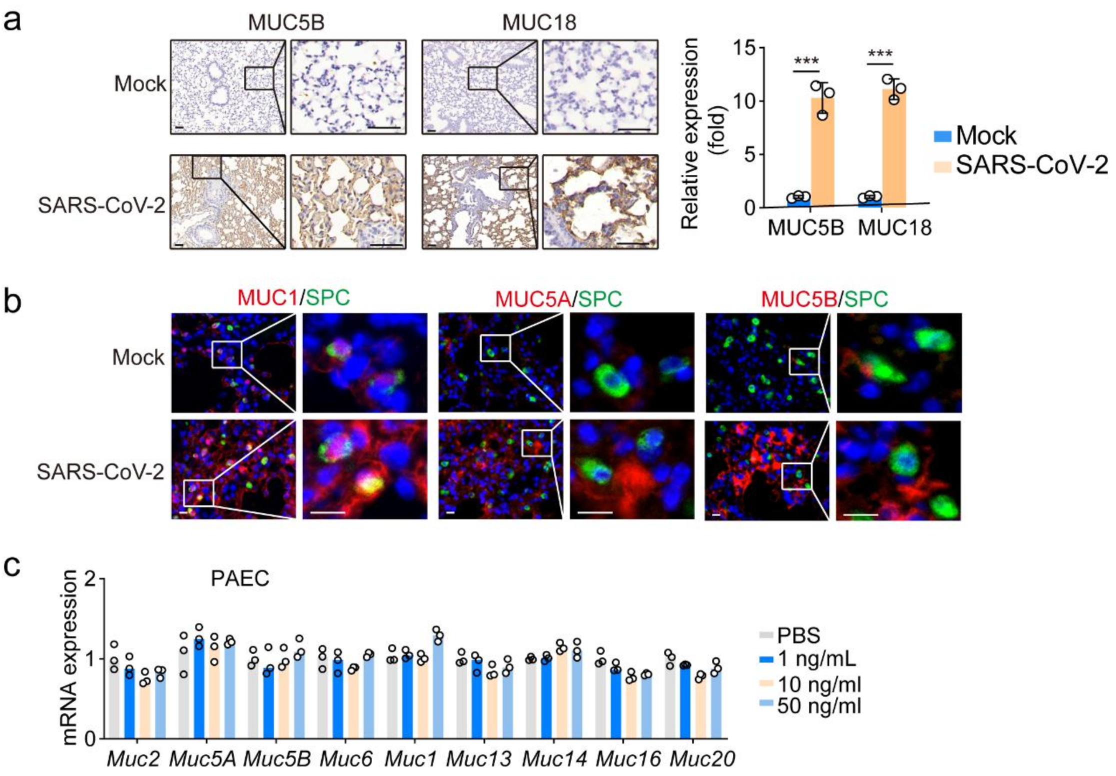

Fig. S2: SARS-CoV-2 infection upregulated the expression of mucins. a Immunohistochemical staining of mucins 5B and 18 from the lung sections of SARS-CoV-2 infected hACE2 transgenic mice. Scale bars, 50 μm. **b** hACE2 transgenic mice were infected with SARS-CoV-2. The lung sections were performed immunofluorescence staining with anti-mucins 1, 5A or 5B (red color) and SPC (green color) antibodies. Scale bar, 10 μm. **c** Primary alveolar epithelial cells (PAEC) were treated with different doses of IL-6 as indicated. The expression of mucins was determined by real-time PCR. The data represent mean ± SD. Representative images are from 3 mice (**a** and **b**). n = 3 biological independent samples (**c**). *** P<0.001, by two-tailed Student's t test (**a**).
