# Supplementary material for: Mucus production stimulated by IFN-AhR signaling triggers hypoxia of COVID-19
Source: Cell Res. 2020 Nov 6;30(12):1078–87. doi: 10.1038/s41422-020-00435-z (PMC7646495; doi:10.1038/s41422-020-00435-z)
Supplement: Supplementary file 3 — Supplementary Figure S3 [file 41422_2020_435_MOESM3_ESM.pdf]

Fig. S3

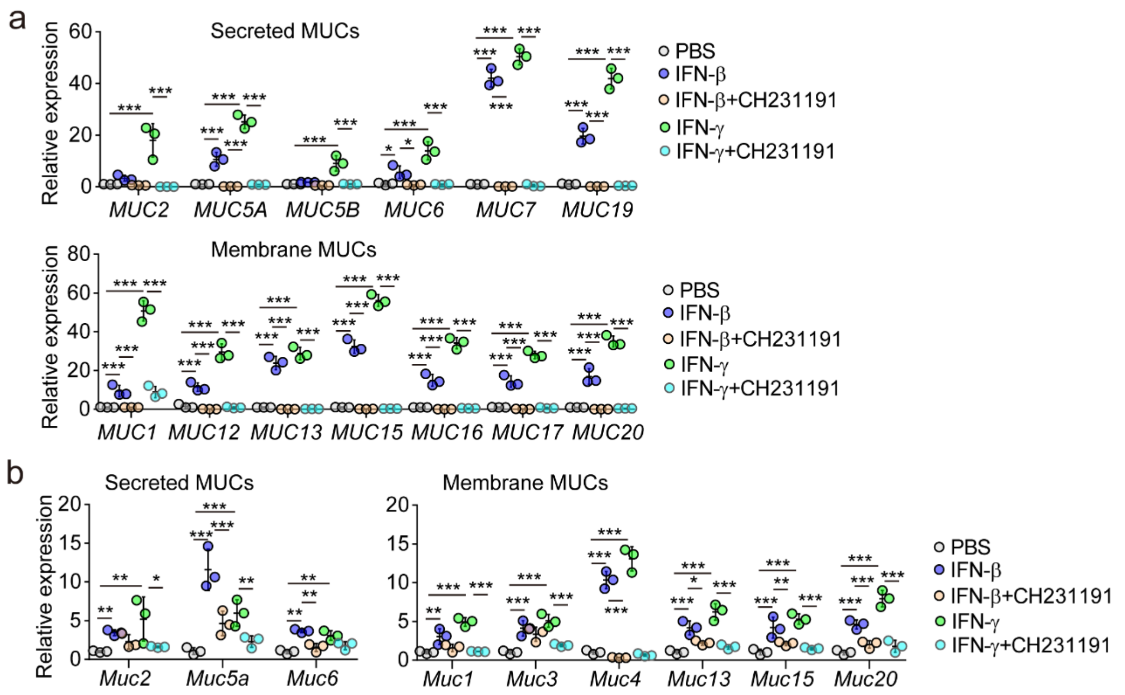

Fig. S3: IFN-β and IFN-γ activate the IDO-AhR pathway to regulate expression of mucins. a BEAS-2B cells were treated with IFN-β (1 ng/mL), IFN-β + CH231191 (2 μM), IFN-γ (10 ng/mL) or IFN-γ + CH231191 for 24 hr. The expression of secreted mucins (*MUCs 2*, *5A*, *5B*, *6*, *7* and *19*) and membrane mucins (*MUCs 1*, *12*, *13*, *15*, *16*, *17* and *20*) was determined by real-time PCR. **b** The same as (**a**), except that PAECs were used. The data represent mean ± SD. In **a** and **b**, n = 3 biological independent samples. * *P*<0.05, ** *P*<0.01, *** *P*<0.001, by one-way ANOVA (**a** and **b**).
