# Supplementary material for: Mucus production stimulated by IFN-AhR signaling triggers hypoxia of COVID-19
Source: Cell Res. 2020 Nov 6;30(12):1078–87. doi: 10.1038/s41422-020-00435-z (PMC7646495; doi:10.1038/s41422-020-00435-z)
Supplement: Supplementary file 4 — Supplementary Figure S4 [file 41422_2020_435_MOESM4_ESM.pdf]

Fig. S4

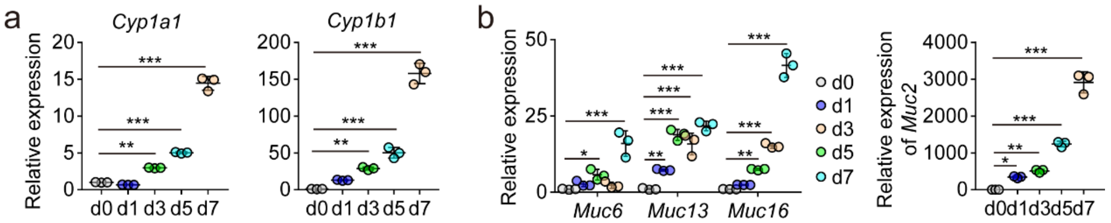

Fig. S4. AhR pathway was activated in SARS-CoV-2 infected hACE2 transgenic mice. hACE2 transgenic mice were infected with SARS-CoV-2 for the indicated time periods. The expression of *Cyp1a1*, *Cyp1b1* (**a**) and *Mucs 2*, *6*, *13* and *16* (**b**) from the lung tissues was determined by real-time PCR. n = 3 mice. The data represent mean ± SD. * *P*<0.05, ** *P*<0.01, *** *P*<0.001, by one-way ANOVA (**a** and **b**).
