# Supplementary material for: Mucus production stimulated by IFN-AhR signaling triggers hypoxia of COVID-19
Source: Cell Res. 2020 Nov 6;30(12):1078–87. doi: 10.1038/s41422-020-00435-z (PMC7646495; doi:10.1038/s41422-020-00435-z)
Supplement: Supplementary file 5 — Supplementary Figure S5 [file 41422_2020_435_MOESM5_ESM.pdf]

Fig. S5

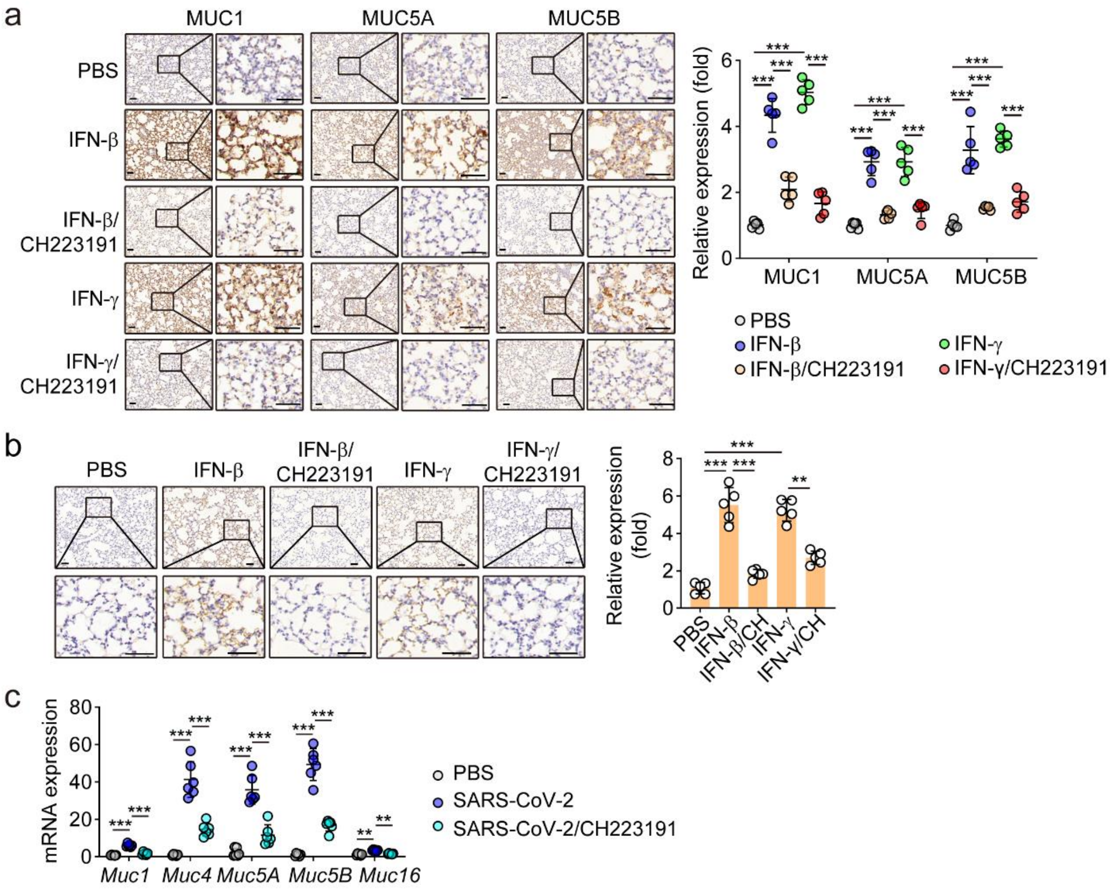

Fig. S5: AhR inhibition decreased the expression of mucins induced by IFNs or SARS-CoV-2 infection. a, b ICR mice were treated with IFN-β (1 μg/mouse), IFN-γ (10 μg/mouse), IFN-β + CH223191 (10 mg/kg) or IFN-γ + CH223191 through the trachea once every day for 4 days. Lung tissues were fixed to perform the immunohistochemical staining with anti-mucins 1, 5A, 5B (**a**) or 2 (**b**). Scale bars, 50 μm. **c** hACE2 transgenic mice were infected with SARS-CoV-2, and then treated with or without CH223191 (10 mg/kg) by tail vein once every day for 5 days (n = 6 mice). The expression of *Mucs 1*, *4*, *5A*, *5B* and *16* from lung tissues was measured by real-time PCR. The data represent mean ± SD. Representative images are from 5 mice (**a**, **b**). ** P<0.01, *** P<0.001, by one-way ANOVA (**a**-**c**).
