# Supplementary material for: Mucus production stimulated by IFN-AhR signaling triggers hypoxia of COVID-19
Source: Cell Res. 2020 Nov 6;30(12):1078–87. doi: 10.1038/s41422-020-00435-z (PMC7646495; doi:10.1038/s41422-020-00435-z)
Supplement: Supplementary file 6 — Supplementary Table [file 41422_2020_435_MOESM6_ESM.pdf]

Table S1: Clinical information of COVID-19 patients

No.	Sex	Age	Diagnosis
1	Male	68	COVID-19
2	Male	82	COVID-19
3	Female	33	COVID-19
4	Male	22	COVID-19
5	Female	37	COVID-19
6	Male	68	COVID-19
7	Female	66	COVID-19
8	Female	63	COVID-19
